# Leveraging Physical Literacy in Schools to Enhance Meaningful Physical Activity Experiences: Mixed‐Methods Findings From an Extracurricular Intervention

**DOI:** 10.1111/josh.70287

**Published:** 2026-11-10

**Authors:** Louisa Schmittwilken, Katharina Pöppel, Anne Williges, Sophia Ryll, Johannes Carl

**Affiliations:** ^1^ Institute of Physical Education and Sport Sciences University of Osnabrück Osnabrück Germany; ^2^ Institute of Sport Science, School IV ‐ School of Humanities and Social Sciences Carl von Ossietzky Universität Oldenburg Oldenburg Germany; ^3^ Stiftung Aktion Hilfe für Kinder Bremen Germany; ^4^ Department of Sport Science and Sport Friedrich‐Alexander‐Universität Erlangen‐Nürnberg Erlangen Germany; ^5^ Deakin Institute for Physical Activity and Nutrition (IPAN) Deakin University Burwood VIC Australia; ^6^ Department of Sport and Human Movement Science University of Vienna Wien Austria

**Keywords:** extracurricular, intervention, mixed‐methods design, PA promotion, physical education, physical literacy

## Abstract

**Background:**

Physical literacy (PL) is a holistic framework designed to facilitate lifelong physical activity. As PL implementations remain limited in Germany, the PLACE (“Physical Literacy and Childhood Enrichment”) study evaluated an extracurricular PL‐based intervention to promote physical activity among primary school children in Bremen, Germany.

**Methods:**

Using an embedded mixed‐methods design, children's perceptions of the PL‐based intervention were compared with standard PE. Data were gathered through group interviews (*n* = 40). Quantitative preferences were analyzed using binomial tests, and qualitative insights were derived through content analysis.

**Results:**

While no differences emerged regarding “social interactions,” “improvement of motor skills and abilities,” and “teaching and classroom management” (*z* ≤ 1.67, *p* ≥ 0.095), the PL intervention outperformed standard PE in “cognitive activation” (*z* = 3.68, *p* < 0.001) and “affective approach to sport” (*z* = 2.47, *p* = 0.014). Qualitative data underscored the children's appreciation for cognitive stimulation and opportunities for “voice and choice.”

**Implications for School Health Policy, Practice, and Equity:**

Integrating holistic PL principles into schools can enhance cognitive and affective engagement, complementing standard PE curricula and informing policies for lifelong physical activity.

**Conclusion:**

The novel PL‐based approach is perceived more favorably by children than standard PE, suggesting its potential to provide meaningful physical activity experiences.

## 1 Introduction

As physical inactivity marks an important risk factor for developing non‐communicable diseases [1, 2], promoting physical activity is essential [3]. Despite the benefits of an active lifestyle, including the prevention of cardiovascular diseases and improved general well‐being [2, 4], global analyses indicate a rising prevalence of inactivity [5, 6, 7, 8]. Worldwide monitoring reveals that 31% of adults and 81% of adolescents do not meet global recommendations [2]. Given the importance of childhood for motor development [9, 10], health status [11, 12], and the links to social and individual values such as cooperation and self‐concept [13, 14] young people should be the focus of efforts to promote active lifestyles. Promoting activity at a young age is critical for sustainability, as early lifestyle patterns often carry over into adulthood [15, 16]. This global interest in fostering lifelong activity is reflected in the growing emphasis on physical literacy (PL) within policy documents aimed at creating a more active society, particularly within school contexts [2, 17, 18, 19].

### 1.1 Physical Literacy

PL combines physical, cognitive, and affective domains and is defined “as the motivation, confidence, physical competence, knowledge and understanding to value and take responsibility for engagement in physical activities for life” [20, 21]. Despite slight cultural variations in conceptualization, the general definition remains the foundation of the concept [22, 23]. The integratively oriented domains and philosophical assumptions grounded in monism, existentialism, and phenomenology illustrate its holistic character and individual orientation, valuing all people regardless of their abilities and environment [20, 24, 25, 26, 27, 28]. While PL has advanced internationally [20, 29, 30], the situation in European countries is still very heterogeneous and needs to be based on broader scientific research [31]. According to the UNESCO QPE guidelines, cultivating PL should be a central aim of quality PE [18], making the lack of systematic engagement in Germany a significant gap. Although theoretical correspondences between PL and established German curricular principles (e.g., “competencies”) exist, their consistent enactment in teaching practice remains unclear [32, 33]. While school‐based PL interventions are increasingly recognized internationally, those explicitly valuing the holistic nature of the concept are rare [34], and existing study designs are often highly heterogeneous [30, 34, 35]. Based on these current gaps and the incomplete discourse of PL in the German context in general [36], we conducted the PLACE (“Physical Literacy and Childhood Enrichment”) study, focusing on both the geographically under‐researched region of Germany and the insufficiently holistic nature of existing PL interventions.

### 1.2 The PLACE Study

Against this backdrop, the PLACE study introduced an extracurricular PL intervention to the German school setting [37]. To create a person‐centered atmosphere without pressure regarding grades, the intervention was intentionally located outside regular PE schedules. Nevertheless, the study considered children's experiences in relation to their familiar PE experiences to provide an educational point of reference. As the German PL discourse is in its early stages and lacks a shared national understanding, we based our study on the Australian PL Framework (APLF) [38], which incorporates physical, cognitive, and psychological/affective domains, adding a fourth social domain [39]. The first two procedural aims of the PLACE study—(a) developing an age‐appropriate PL intervention and (b) implementing it in German primary schools—were achieved during the pilot cycles [37, 40, 41]. The final aim (c), to evaluate the intervention's effect on the participating children, is the subject of this article.

## 2 Methods

### 2.1 Participants

Following the Physical Literacy Interventions Reporting Template (PLIRT) [42] (Supporting Information file S1), the main study involved approximately 200 children across 10 groups between August 2023 and February 2024. A subsample of *N* = 40 children (*n* = 7 third graders, *n* = 33 fourth graders) from four groups across three primary schools was selected to balance field feasibility with the depth required for a mixed‐methods approach. Data saturation was reached through nine focus group interviews [43], prioritizing in‐depth exploratory understanding over statistical generalizability. Purposive sampling ensured the subsample reflected the overall cohort's diversity regarding age, coaches, and implementation settings (regular classes vs. afternoon programs). To maintain a child‐friendly, low‐barrier design and optimize the pre‐interview consent process, demographic data were collected at the group level; however, the subsample mirrored the diverse socioeconomic profiles of the participating schools. Notably, all schools were part of the Federal Ministry of Education and Research's “Startchancen” program, which supports schools with a high proportion of socially disadvantaged pupils. The children's cultural backgrounds were particularly diverse, and one child participated with a personal assistant due to behavioral challenges. Participation required written parental/guardian consent, verbal consent from the child, and attendance in at least 10 intervention sessions to ensure sufficient exposure.

### 2.2 Instrumentation

The PLACE study began with two pilot cycles to undertake adjustments for the target group [37]. The interventions spanned three school terms, involving over 500 children across eight primary schools in Bremen, Germany. Pilot insights informed minor content adjustments [41]. However, the initial evaluation approach proved problematic: all three PL instruments were psychometrically unsatisfactory after translation into German and demonstrated substantial ceiling effects at baseline. Furthermore, children and coaches reported cognitive overload and frustration with the assessments [40]. These validity challenges and participant feedback prompted a fundamental reconsideration of the evaluation approach [40]. Consequently, the main phase adopted a child‐centered mixed‐methods approach, preserving the study's quantitative orientation while remaining responsive to the participants' needs. Rather than measuring PL as a static outcome, this approach evaluated the enabling conditions required to foster PL development. An embedded design allowed for an intrapersonal comparison of children's perceptions of regular PE and the PL‐based intervention, with qualitative data contextualizing the quantitative findings [44, 45]. Comparisons were based on literature‐derived quality PE criteria, providing a standardized benchmark across different settings [46, 47, 48, 49, 50, 51]. Although not direct components of the PL framework, they are conceptually aligned, as high‐quality PE is a pedagogical prerequisite for PL development [18]. To ensure suitability for the target group and avoid national bias, five criteria were selected: (1) social interactions; (2) improvement of motor skills and abilities; (3) cognitive activation; (4) affective approach to sport; and (5) teaching and classroom management. Supporting Information File S2 represents a full comparison of the quality PE criteria.

### 2.3 Intervention

The intervention addressed all four APLF domains by formulating goals related to “physical,” “psychological/affective,” “cognitive,” and “social” aspects. We emphasized respect for individual abilities and integrated participatory elements (“voice and choice”), aligning with the UN Convention on the Rights of the Child (UNCRC; Article 12) [52] and Self‐Determination Theory (SDT), which advocates for autonomy‐supportive environments to enhance intrinsic motivation and affective engagement [53]. The program was delivered either during daily after‐school hours (mixed groups; 12–18 children) or as extracurricular periods within the school day (regular school classes; 20–24 children). Attendance was compulsory and weekly, mirroring the institutionalized character of regular PE. The 14‐week program consisted of 60–90 min sessions delivered by one PL coach (holding bachelor's degrees in physical education or health management, sports experience, and trainer licenses). To ensure high fidelity, the program was iteratively developed through pilot phases [41]. During implementation, coaches adhered to the planned structure and core PL principles, monitored through regular coordination meetings and session protocols [41]. The main study involved 10 groups across eight primary schools; while these schools participated in pilot cycles, no classes in the main study had prior PL intervention exposure. Each session included 10–15 min of free play, 30–40 min of structured activities (games, exercises, or station runs), and concluded with a child‐selected game (15–20 min) and a closing ritual. Table 1 provides an overview of the intervention content.

**TABLE 1:** Structure and content of the 14‐week main‐phase PLACE intervention: session focus, goals, and PL domains.

			Physical literacy domain			
Session	Focus	Main goal	Physical domain	Cognitive domain	Psychological domain	Social domain
1	Get to know	Explanation of the study	Playful movements	Session rules and general rules in sport	Building relationships; participation	Get to know each other
2–5	Parkour I	High physical activity time; experience variety of movement	Agility; overcoming obstacles	Meaning of movement in everyday life	Recognition of personal skills	Respect toward others and their abilities; integrity
	Ball games I	Improving individual ball control (object control)	Object control in various ball games	Winning and losing in sport	Individual progress and confidence in object control	Fair play; playing cooperatively and competitively
	Acrobatics	Experience different acrobatic formations	Stability; balance	Muscle tension and relaxation	Emphasis on courage; self‐awareness and trust	Communication; collaboration
	Free session	Reflection of popular games	Various movements	Modification of games	Contribute own ideas/whishes	Playing together
6–10	Endurance games	Pacing oneself	Endurance and speed	Pulse knowledge	Self‐regulation	Support others; relationships
	Frisbee	Learning something new	Object manipulation; throwing and catching a frisbee	Fair play in sport (repetition)	Motivation; fun	Trying new things together
	Gymnastics	Creativity in movement	Strength; flexibility; balance	Variety of gymnastics	Risk‐taking	Collaboration
	Racket sport	Hand‐eye coordination	Handling rackets	Benefits of regular activity	Confidence in handling equipment	Playing cooperatively and competitively
	Free session	Reflection and adaptation of popular games	Various movements	Modification of games	Contribute own ideas/wishes	Playing together
11–13	Ball games II	Improving specific object control	Object control in various ball games	Winning and losing in sport	Individual progress and confidence in object control	Fair play; playing cooperatively and competitively
	Scuffling	Regulate strength	Strength; stability	Rules for scuffling	Self‐regulation; appropriate use of strength	Respect others and their limits
	Parkour II	Development of proper use of movements in the context of parkour	Jumping; running; overcoming obstacles	Parkour as lifestyle; connection to outdoor practice	Recognition of personal skills	Respect and integrity
14	Post evaluation	Evaluation	Playful movements	Key take‐home message	Self‐assessment	Farewell and reflection

### 2.4 Procedure

A total of nine group interviews were conducted by an independent person unknown to the children and lasted on average 15:54 min (9:20–27:07). The interview format supports value‐free communication, reduces adult‐child hierarchies through the children's numerical majority [54], is familiar to children, and helps capture children's feelings, opinions, and reactions [55]. Framed by an introduction and open‐ended conclusion, the interview focused on intrapersonal comparisons between the intervention (TRYZE) and standard PE from the children's perspective. Following the mixed‐methods approach, data were collected in two steps for each predefined criterion. First, children indicated their preference between TRYZE (blue sticker) or regular PE (yellow sticker) for each criterion. To ensure unbiased, individual responses, the interviewer (green) read the statements aloud while children (purple) retrieved stickers from spaced cups and placed them on the sheet (Figures 1 and 2). Subsequently, children explained their choices. This interactive, movement‐based approach was used to maintain attention and encourage participation compared to traditional formats [55, 56].

**FIGURE 1:**
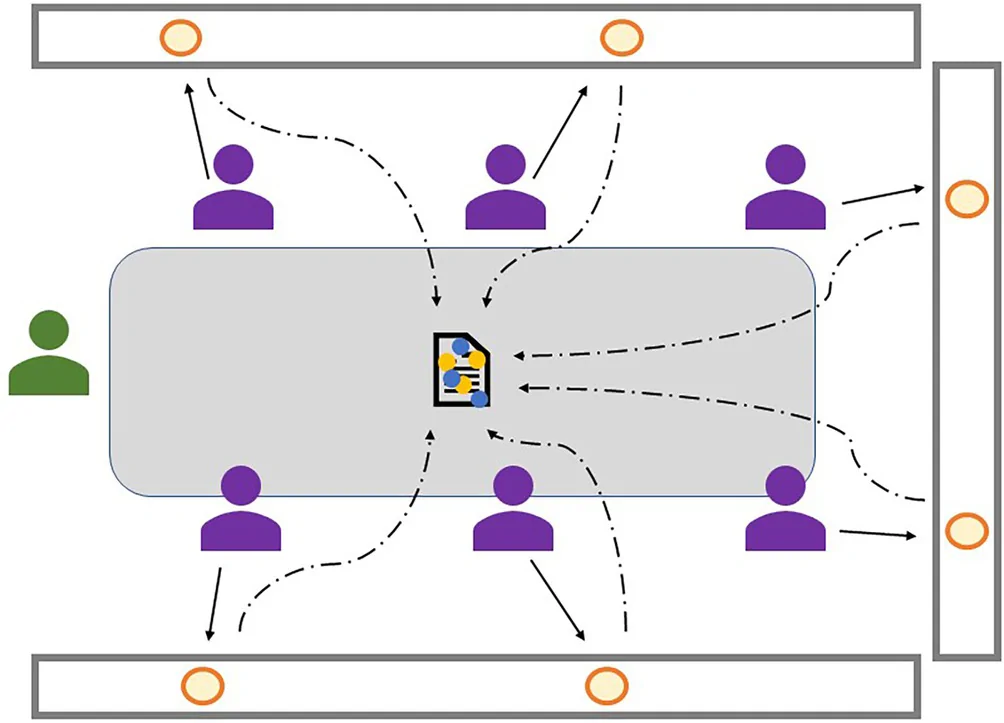
An example of how the interview situations are set up.

**FIGURE 2:**
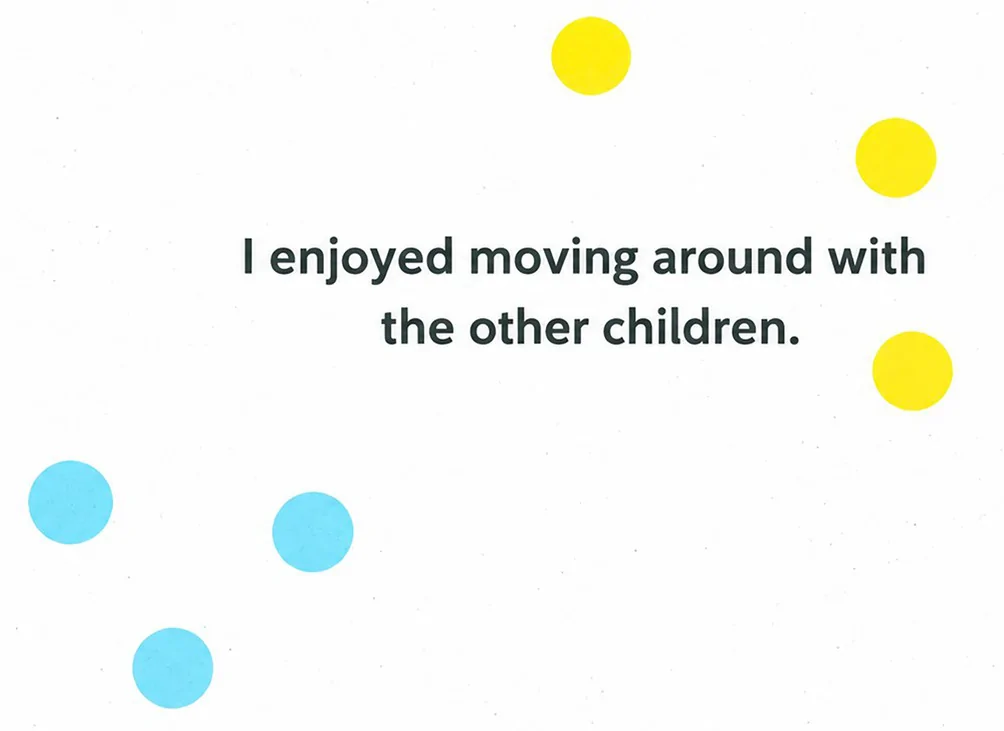
Sample answer sheet regarding the statement “I enjoyed moving around with the other children,” relating to the category of social interactions with six stickers.

### 2.5 Data Analysis

Collected quantitative and qualitative data were analyzed deductively, based on the five selected criteria of quality PE. An embedded design with simultaneous data collection was used, with qualitative data providing a complementary perspective to the quantitative results [44, 45, 57]. Quantitative data were retrieved from the distribution of children's dots located on either the PL intervention or regular PE side. Where no preference was indicated, an equal distribution was assumed. A two‐sided binomial test examined whether dots significantly accumulated on one side. Given the dichotomous nature of the preference data (TRYZE vs. regular PE), the binomial test was selected to examine whether the observed proportion of preferences differed significantly from the null expectation of equal preference (50%). Because each quality criterion was analyzed separately, each child contributed one dichotomous preference decision per criterion, which formed the basis for the respective binomial analysis. *Z*‐scores and corresponding *p* values (significance level *p* < 0.05) were calculated to assess the likelihood of false differences. Confidence intervals at the 95% level (CI: 95%) were presented around the percentage values of the strongest preference option. Accordingly, the quantitative findings should be interpreted as reflecting children's preference distributions across the selected quality PE criteria rather than objective indicators of learning, PL, or intervention effectiveness. Qualitative interviews were transcribed and anonymized following established rules [58]; with analysis supported by MAXQDA (2024). To filter and summarize relevant content, we followed Mayring's seven‐step “process model of deductive category application (structuring)” [59]. After defining the research subject, question and theoretical framework (Step 1), and developing a theory‐based category system (Step 2), the first author created a coding guide including definitions, anchor examples, and coding rules (Step 3). A research team then refined the guide, acting as “critical friends” [60], challenging assumptions and clarifying coding decisions (Step 4). The first author analyzed all interviews (Step 5), while the fourth author independently reviewed ambiguous passages for team discussion (Step 6). Finally, the first author organized the coding into subtopics and integrated them with the quantitative findings (Step 7). As categories were consistent across both analyses, integration was straightforward. The full process and coding guide are detailed in Supporting Information files S3 and S4.

## 3 Results

### 3.1 Overview

The quantitative results are summarized in Table 2, followed by a description of the qualitative findings. We generated a total of 148 codes. In line with the research goals, we focus on the 135 codes that could be clearly assigned to either the TRYZE intervention or regular PE. The 13 codes that could not be clearly assigned to one of the two groups (no significant accumulation in any of the five categories) were excluded from the subsequent analysis. In general, the children referred more frequently to TRYZE (96 TRYZE; 39 PE). Apart from six positively (4 TRYZE; 2 PE) and nine rather negatively rated statements (7 TRYZE; 2 PE), the majority of the interview statements are either neutral (72) or rather positive [48]. Figure 3 gives an overview of the overall responses.

**TABLE 2:** Quantitative results examining the preference of the PL intervention versus regular physical education.

		Preference of intervention				
Category	Formulation	PL intervention	Regular PE	Indecisive	*z*	*p*
Social interactions	I enjoyed moving around with the other children	23	13	4	1.67	0.096
2 Improvement of motor skills and abilities	I have become better at sport	19	15	6	0.686	0.493
3 Cognitive activation	I learned a lot about exercise and my body	31	8	1	3.68	**< 0.001**
4 Affective approach to sport	I looked forward to the lesson every week	26	11	3	2.47	**0.014**
5 Teaching and classroom management	The lessons were well led	20	15	1	0.845	0.398

*Note:* Two‐sided test; PE = Physical Education. Bold indicates a statistically significant preference of the PL‐intervention over regular PE.

**FIGURE 3:**
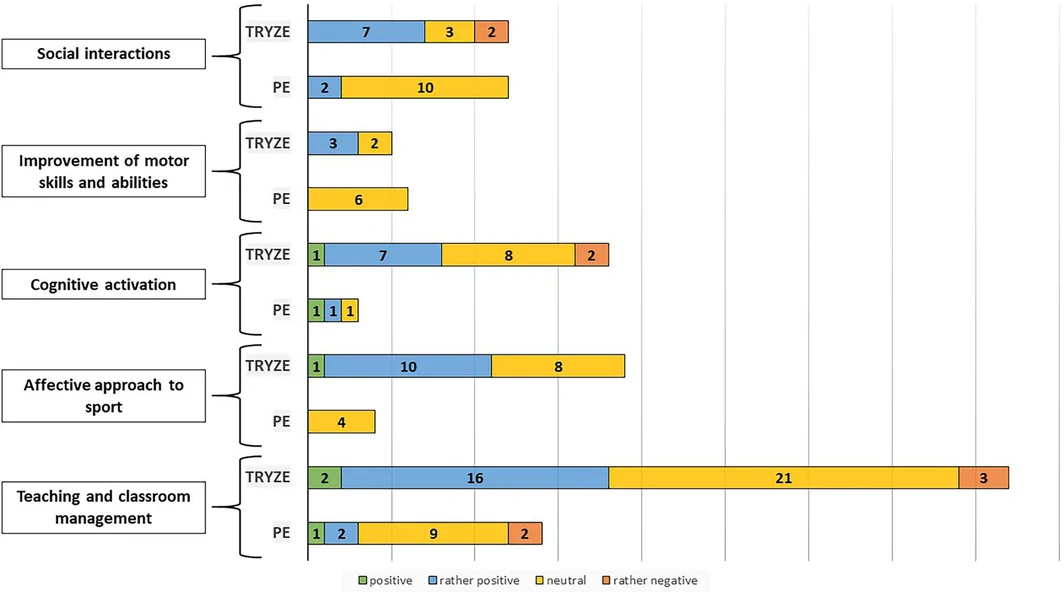
Overview of all responses to the five selected criteria divided in TRYZE and PE, categorized as “rather positive,” “positive,” “neutral” and “rather negative.”

### 3.2 Social Interactions

When analyzing children's voting patterns, the assumption of equal distribution between both conditions was maintained for the “social interactions” category (TRYZE: 23/36 preferences, 63.9%, 95% CI: 46.2%–79.2%; *z* = 1.67, *p* = 0.096). Accordingly, the number of preferences did not significantly differ between TRYZE (*n* = 23) and regular PE (*n* = 13). Looking more closely at the coded passages in the “social interactions” (*n* = 24) category, we identified three overarching areas verbalized by the children: (a) general aspects at the child's level, (b) the behavior of the respective teacher, and finally (c) links to explicit content or to the basic structure of the lesson. *General aspects at the child's level* were evenly distributed between the two comparison dimensions; for example, the children were more polite to each other, or the specific group constellation worked better (newly formed group in TRYZE/well‐known group in PE). In addition, there were also statements about arguments among the children and a generally higher noise level in the lessons. Although some children acknowledged this for regular PE, this case was made more frequently in relation to the TRYZE lessons. In relation to the *behavior of the respective teacher*, some children noted that the class felt like “chaos” when the class teacher was absent, while others criticized their regular PE teacher for not allowing them to comfort injured classmates. Regarding *links to explicit content or to the basic structure of the lesson*, children mentioned that either regular PE or TRYZE included more team games and cooperative structures. Finally, they recommended the opportunity for free play and group choice—attributed to TRYZE—as beneficial for social interactions:

### 3.3 Improvement of Motor Skills and Abilities

Likewise, the assumption of equal distribution between both conditions was maintained for the “improvement of motor skills and abilities” category (*z* = 0.686, *p* = 0.493). Children preferred TRYZE in 19 of 34 comparisons (55.9%, 95% CI: 37.9%–72.8%), indicating no statistically significant preference over regular PE (*n* = 15). Considering the qualitative data (*n* = 13), most coded passages can be assigned to *structural aspects and specific lesson content*. Apart from this topic, further individual statements referred to either TRYZE or regular PE, such as clearer explanations by the TRYZE teacher or the goal‐oriented nature of regular PE (regarding the sport festival)—both seen as ways to improve motor skills. Most reasons, however, focused on the character of the content: While some children valued free play and self‐paced exploration in TRYZE, others appreciated the longer duration and more sport‐specific focus of regular PE.

### 3.4 Cognitive Activation

The assumption of equal distribution between both conditions was not met for the “cognitive activation” category (*z* = 3.68, *p* < 0.001). Children preferred TRYZE in 31 of 39 comparisons (79.5%, 95% CI: 63.5%–90.7%), marking a statistically significant preference over regular PE (*n* = 8). Within the qualitative findings (*n* = 21), except for two references regarding an improved explanation by the respective teacher (as previously mentioned), the majority of coded segments related to lesson structure and, in particular, to cognitively activating elements. A frequently mentioned aspect was the presence of extended and in‐depth discussions and reflections, which were observed equally in both settings. Notably, nearly half of the coded content within this category consisted of detailed accounts by children describing their specific experiences in TRYZE, as the following statements exemplify:

[B]efore we moved was to measure our (.) pulse for °one minute° how often it beat and then we were supposed to move really hard (.) and then we were supposed to count and then it was just more (.) and yes, that's when we learnt a bit more about physical education (MOAU_1, 51; translated L.S.)

[W]ell, at TRYZE we actually talked about it […] for example, whether your arm muscles (.) get stronger from rowing or from running or something like that (.) and we also talked a lot about how much physical activity you need and things like that (MIKA_2, 62; translated L.S.)

### 3.5 Affective Approach to Sport

The assumption of equal distribution of preferences between both conditions was not maintained for the category of “affective approach to sport” (*z* = 2.47, *p* = 0.014). Children preferred TRYZE in 26 of 37 comparisons (70.3%, 95% CI: 53.0%–84.1%), representing a statistically significant preference over regular PE (*n* = 11). Based on the qualitative data, four themes were derived from the interview codes (*n* = 23): (a) the structure of the lesson, (b) the explicit content, (c) the respective teacher and, finally, (d) the behavior between the children. Regarding the *structure of the lesson*, aside from a generally longer playing time in TRYZE, most children commented positively on TRYZE's basic lesson plan, with freely organizable elements and a fixed topic—highlighting increased opportunities for participation and co‐decision making:

[A]nd what I always found so cool […] we first had playtime […] then we had some topic (.) where we played and the last thing was that a child was always allowed to draw a game of their choice and then we were allowed to play it (MIKA_2, 179; translated L.S.)

In terms of *explicit content*, children's explanations focused on novelty, changing areas, and individual preferences, which are often linked to TRYZE:

I prefer TRYZE too (.) because right at the start (.) we'd been given these little pieces of paper where we wrote down a game – everyone wrote down their own game – […] if we still had time, we'd pick out a game (MOAU_1, 61; translated L.S.)

At the level of *the respective teacher*, rigor emerged as a key theme, although often described ambiguously. In both settings, some children preferred a more controlling teaching style, while others preferred less. The final, rarely mentioned theme concerned to *the behavior between the children* and corresponds to the previously addressed “social interactions” category. Children linked affective dimensions of sport to social dynamics (e.g., politeness), with positive associations being more frequently attributed to regular PE than to TRYZE.

### 3.6 Teaching and Classroom Management

Finally, equal distributions between both conditions were assumed for the “teaching and classroom management” category (*z* = 0.845, *p* = 0.398). Children preferred TRYZE in 20 of 35 comparisons (57.1%, 95% CI: 39.4%–73.7%), indicating no statistically significant preference over regular PE (*n* = 15). Within the category “teaching and classroom management,” the statements (*n* = 56) can be divided into three areas: (a) the basic structure of the lesson, (b) the teacher's behavior and actions, and (c) the explicit content of the lesson. Regarding the *basic structure of the lesson*, most children highlighted the positively perceived mixed lesson structure. Furthermore, the weekly rotation of topics (TRYZE) versus the in‐depth exploration over several weeks (PE) was discussed controversially, with varying preferences. Concerning the *teacher's behavior and actions*, rigor emerged as the most frequent aspect: While some children favored teachers' greater rigidity and assertiveness, others emphasized the benefits of a more relaxed approach. Most commonly the TRYZE teacher was seen as less restrictive. Less frequent comments referred to the TRYZE teacher's clearer explanations and the reassuring nature of the teachers in regular PE, which contributed to calmer behavior and fewer boundary‐testing incidents. In terms of the *explicit content of the lesson*, the children associated team‐based games and regular cognitive‐activation reflection periods with either TRYZE or regular PE.

## 4 Discussion

The PLACE study investigated an age‐appropriate, holistic PL‐based extracurricular physical activity intervention in German primary schools. Using an embedded mixed‐method design, the intervention (TRYZE) was compared with standard PE across five literature‐derived quality PE criteria. While the sticker‐based voting procedure was not an objective measure of PL development, it provided developmentally appropriate, nuanced insights into children's experiences. Children perceived TRYZE as superior to regular PE regarding “cognitive activation” and “affective approach to sport,” likely due to its cognitively stimulating, child‐centered, and co‐creative structure. These findings suggest that PL‐based interventions may be perceived to address specific quality PE criteria more comprehensively; specifically, the preference for choice and co‐creation reflects the affective PL domain by nurturing motivation and engagement, a finding that aligns with previous PL‐based interventions [61, 62]. The children's reflections on cognitive stimulation align with the cognitive domain of the APLF, as it is closely linked to reasoning and content knowledge, as the children mentioned [38]. No significant differences emerged between PE and TRYZE regarding “social interactions,” “improvement of motor skills and abilities,” and “teaching and classroom management,” indicating these criteria were similarly fostered across both conditions. As TRYZE was delivered in an extracurricular setting and potential novelty effects of content as well as the coaches' behavior remains unclear, these results may complement rather than replace existing PE approaches. At the European level, the implementation of the PL approach remains heterogeneous and requires further research [31, 63], while PL has only hesitantly gained traction within the German sport science discourse [36]. Alongside emerging theoretical engagement and assessment developments [64, 65, 66], the PLACE study represents the first evaluation of a German PL‐based intervention and indicates promising child‐reported preferences. While many school‐based interventions target primary school children, few focus on an extracurricular setting [61, 67], and even fewer address all PL domains holistically [35, 68]. Existing interventions emphasizing physical and cognitive domains have shown improvements in balance and participation [67]; our findings extend this evidence by indicating benefits in the cognitive and affective domains reported by the participating children within the German context. Despite initial challenges in obtaining valid, child‐centered data [40], the final evaluation design successfully engaged children through verbal and non‐verbal methods. The preference‐based quantitative approach provided a developmentally appropriate means of comparing children's evaluations of both educational experiences. Rather than demonstrating intervention effects, the binomial analyses identified whether children systematically preferred one educational experience over the other for each quality criterion. The accompanying qualitative data therefore played a crucial role in contextualizing these preference distributions.

### 4.1 Implications for School Health Policy, Practice, and Equity

These initial practice‐oriented findings highlight contextually responsive practices that may enrich the understanding of quality PE, particularly through co‐creative learning, cognitive engagement, and child‐centered pedagogy. The preference for the PL‐based intervention, especially regarding “voice and choice,” aligns with Self‐Determination Theory (SDT). By fostering autonomy, competence, and relatedness, the intervention acted as an autonomy‐supportive environment [53]. This likely shifted the children's perception from a mandatory requirement to a self‐determined experience, thereby enhancing their intrinsic motivation and affective engagement. Given that fostering PL is considered a foundation for lifelong physical activity [18, 19], a PL perspective may complement existing approaches within the German PE discourse and practice. As many school‐based physical activity programs tend to be discontinued after the initial research‐led implementation, sustaining TRYZE in Bremen would be desirable. Future efforts could include adaptation to other age groups and federal states, alongside evaluation using validated assessment tools. Research should also examine the long‐term effects of PL‐based interventions on children's actual physical activity outcomes and supportive structural conditions. In line with the holistic and subject‐centered nature of PL, future evaluations may benefit from triangulating children's perspectives with teacher observations or objective assessments. Given that TRYZE was conducted during extracurricular hours, it would be beneficial to incorporate PL principles into regular PE to isolate the effects of the framework itself, potentially fostering children's PL in a more systematic and sustainable manner. To further bridge the gap between theory and practice in German PE and given teacher's pivotal role in fostering PL, future research should build upon international evidence by systematically incorporating the perspectives of PE teachers [69].

### 4.2 Strengths and Limitations

To the best of our knowledge, this is the first school‐based PL intervention in Germany explicitly aligned with the four dimensions of the APLF [38]. A further strength is the embedded mixed‐methods design, combining quantitative and qualitative data. Notwithstanding these strengths, several limitations exist. First, the comparison between the extracurricular PL intervention and standard PE may not be entirely equivalent; the novelty of the intervention and the distinct professional profiles of the coaches—compared to regular teachers—might have influenced students' engagement and outcomes. However, many children's explanations specifically highlighted autonomy, voice, and choice, which are theoretically consistent with PL‐informed pedagogy [70]. Given the exploratory design, the quantitative component was not intended for population‐wide generalization, but to identify systematic preferences within the sample. Furthermore, the regional focus (Bremen) and a lack of detailed demographic data limit the generalizability of the findings and preclude a detailed examination of inclusion and equity (e.g., socioeconomic or migration background). Such constraints are inherent to exploratory research in a field where standardized tools are still emerging [71]. Additionally, the disproportionate number of references to TRYZE (96 vs. 39 coded statements for PE) and potential interviewer or group influences should be considered. Finally, the study does not provide long‐term effects or direct changes in PA levels. Nevertheless, as the first PL intervention within the German school system, these results provide a valuable orientation for integrating PL practices and inspire further research to harmonize the international PL approach with the dominant PE framework in Germany.

## 5 Conclusion

The present article contributes to a deeper understanding of the implementation and perceived effects of an extracurricular PL‐based intervention in German schools. An intrapersonal comparison with standard PE suggests that a PL‐based intervention is preferred from the children's perspective, particularly regarding the cognitive‐enhancing content and the lesson structure, which combines predefined elements with opportunities for participation and co‐creation. This study suggests that PL‐based programs implemented in school settings may serve as promising approaches to fostering learning experiences that are conceptually linked to lifelong engagement in physical activity. The high level of acceptance among the children suggests that a holistic approach to PL could emerge as a promising strategy for enabling meaningful physical activity experiences in schools, thereby contributing to the effort of counteracting decreasing opportunities for movement and establishing lifelong active lifestyles. Further research is needed to develop valid assessment tools for PL in Germany and to investigate the long‐term effects on children's actual physical activity behaviors and health outcomes.

## Funding

This work was supported by the Stiftung Aktion Hilfe für Kinder (TRYZE.Active; approval 2022‐02‐23).

## Ethics Statement

The studies involving humans were approved by the Ethics committee of the University of Oldenburg, Germany (sign Drs.EK/2022/057). The studies were conducted in accordance with the local legislation and institutional requirements.

## Consent

Written informed consent for participation in this study was provided by the participants' legal guardians.

## Conflicts of Interest

The authors declare no conflicts of interest.

## Supporting information


**Supporting Information file S1:** The PLIRT checklist.

**Supporting Information file S2:** Overview of high‐quality PE criteria.

**Supporting Information file S3:** Seven‐step process model of deductive category application (structuring) (Mayring, 2022).

**Supporting Information file S4:** Coding guide.

## Data Availability

The data that support the findings of this study are available from the corresponding author upon reasonable request.
