# Supplementary material for: Leveraging Physical Literacy in Schools to Enhance Meaningful Physical Activity Experiences: Mixed‐Methods Findings From an Extracurricular Intervention
Source: J Sch Health. 2026 Nov 10;96(11):e70287. doi: 10.1111/josh.70287 (PMC13750100; doi:10.1111/josh.70287)
Supplement: Supplementary file 1 [file JOSH-96-0-s001.docx]

Subject, research question, theoretical background

Comparison of PL-based interventions and regular PE alongside (inter)national criteria of high-quality PE

Theory-based definition of categories (nominal or ordinal)

Nominal designed category system (based on the selected criteria of high-quality PE)

Theory-based formulation of definitions, anchor examples, and coding rules for each category, compilation into a coding guide

Initial version of a coding system along interviews 1-3 including definitions, anchor examples, and coding rules

Coding of a first text section; revision of the categories and the coding guide

Revision of the initial version based on the first interview (within a research group)

Final material review; assignment of categories to text passages

Final material review of all interviews on the basis of the final category system

Intercoder compliance test

Separate coding of all interviews by a second person

Discussion of ambiguous coded passages (within a research group)

Evaluation; possibly quantitative analysis (e.g. frequencies)

Systematic organization into subtopics based on the previous coding

Dark blue: Process model of deductive category application (structuring) (Mayring, 2022, p. 97) / Light blue: Own approach along the defined steps

Mayring, P. (2022). *Qualitative Inhaltsanalyse: Grundlagen und Techniken* (13. Neuausgabe). Julius Beltz GmbH & Co. KG. http://nbn-resolving.org/urn:nbn:de:bsz:31-epflicht-2019387
