# Supplementary material for: Leveraging Physical Literacy in Schools to Enhance Meaningful Physical Activity Experiences: Mixed‐Methods Findings From an Extracurricular Intervention
Source: J Sch Health. 2026 Nov 10;96(11):e70287. doi: 10.1111/josh.70287 (PMC13750100; doi:10.1111/josh.70287)
Supplement: Supplementary file 2 [file JOSH-96-0-s002.docx]

| **Main category** | **Definition** | **Coding rule** | **Anchor example** |
| --- | --- | --- | --- |
| **Social interactions** | In the 'Social Interaction' category, we are interested in statements that emphasise the importance of social interaction between children. Statements on cooperation and basic social interaction are of interest, as are situations that exclude these, such as arguments between children.  The category follows the key question ‘I enjoyed moving around with the other children.’ | The children report on interpersonal aspects within the group, such as cooperative situations and support, as well as arguments and conflicts between individual children or within the group as a whole. The focus is on how the children interact during the lesson (Ex. 1). Statements about cooperation may also be illustrated by specific lesson content or types of play (Ex. 2) | ‘I made it yellow because (.) we didn't have so much arguing and complaining in PE lessons and in TRYZE there was a lot of complaining and arguing and I didn't like that [...]’ (MOLE_3, 60; translated L.S.) (Ex. 1)  ‘It was the same for me in PE lessons (.) we did more:: other things (.) not so many cooperative games (2) and at TRYZE we did more cooperative games’ (MOLE_2, 19; translated L.S.) (Ex. 2) |
| **Improvement of motor skills and abilities** | All statements and aspects relating to the body, fitness, motor skills or (the improvement of) individual abilities and skills play a role within the category of ‘Improvement of motor skills and abilities’.  ‘I have become better at sport (e.g., stronger or faster)’ is the corresponding key question. | These are statements in which children describe improvements in their motor skills gained through the lessons. Specific areas of movement can be mentioned (Ex. 1), as well as more general aspects that do not include a concrete description of physical improvements (Ex. 2).  Additionally, there are statements in which children do not explicitly mention any improvements, but refer to content that specifically concerns the physical level (Ex. 3). | ‘I also think that because we're currently having the topic scuffling (.) and I just think that some people (.) °who weren't so strong have become stronger°’ (MOAU_1, 67-68; translated L.S.) (Ex. 1)  ‘I (.) um (.) I think I got better in PE lessons (.) because we also trained for sports festivals and stuff and that's part of PE lessons and not TRYZE’ (MOLE_3, 38-39; translated L.S.) (Ex. 2)  ‘Because in TRYZE we also moved around (.) we did a lot of parkour’ (MOLE_3, 24; translated L.S.) (Ex. 3) |
| **Cognitive Activation** | The ‘Cognitive activation’ category includes statements that address the expansion of general knowledge or learning opportunities within the lessons in the context of movement, the body or similar areas.  This category can be assigned to the key question ‘I have learnt a lot about movement and my body’. | The children describe lesson content that can specifically be assigned to the expansion of knowledge within the context of the lessons (Ex. 1). They also mention learning successes explicitly (usually using phrases such as ‘I have learnt’), as well as explicit learning occasions and reflection impulses (often using phrases such as ‘we talked about it’), or subjective attributions of meaning or relevance (Ex. 2). | ‘So I learnt when you run so fast that you should do this ((points to the wrist)) and that you should somehow count how much heart pressure you have and yes (.)’ (MOAU_1, 83-85; translated L.S.) (Ex. 1)  ‘Yes (.) so with TRYZE we really talked about it (.) we also made cards (2) where, for example, the arm muscles (.) get stronger from rowing or rather from running or something (.) and we also talked a lot about how much exercise you need and so on and that's why I decided in favour of TRYZE’ (MIKA_2, 61; translated L.S.) (Ex. 2) |
| **Affective approach to sport** | Statements in the 'Affective approach to sport' category highlight the presence of content designed to enhance the enjoyment and motivation of lessons.  ‘I looked forward to the lesson every week’ is the corresponding key question. | The children outline aspects of (pre-)enjoyment, fun, or motivation in relation to the lessons. Phrases such as ‘I looked forward to it’ or ‘I had fun’ are an indication of this category. The affective approach is often mentioned in connection with other categories, including lesson and classroom management. This is because affective attributions can be justified with regard to lesson content or the atmosphere, (Ex. 1+2). Double coding is possible and appropriate in these cases. | ‘Well:: (.) I sometimes looked forward to PE lessons (2) but there were also sometimes things (.) that I didn't like so much and at TRYZE I actually always enjoyed things (2) °yes° (2)’ (MIKA_2, 164-166; translated L.S.) (Ex. 1)  ‘I was also looking forward to TRYZE (.) so I was looking forward to it in PE lessons but I wasn't so happy about it (.) because there was always complaining and °that's why I took yellow° (2) # 1#’ (MOLE_3, 60; translated L.S.) (Ex. 2) |
| **Teaching and classroom management** | The category ‘Teaching and class management’ includes aspects that relate to either lesson management or the general atmosphere in the lesson, such as the behaviour of the teacher, interaction with the children, etc.), or relate to structured lesson content, such as recurring elements.  This category was derived from the key question ‘The lessons were well led’. | Coded statements in this category can be coded at two superordinate levels. On the one hand, the focus is on the lesson leader; the children report on behaviour, general appearance, and how they interact with the children, in reference to either the sports teacher or the TRYZE leader (Ex. 1). In some cases, specific names are mentioned, such as Lena, Louisa, or the names of teachers, which are anonymised.  On the other hand, comments on the structure of the lessons are of interest, e.g., recurring features of lessons that the children explicitly assign to one of the two lessons (Ex. 2). | ‘Um, so I think our teacher is stricter in PE lessons and if a child makes nonsense then they are sent to the bench (2) um and here (.) maybe the children (.) didn't listen so well uh and carried on anyway (3)’ (MOAU_1, 155-157; translated L.S.) (Ex. 1)  ‘Well, I think at TRYZE we always had (.) so it was always like this, we always had a programme (.) we always had um play times and then we had a theme and that was actually always a lot of fun’ (MIKA_2, 19; translated L.S.) (Ex. 2) |
