# Supplementary material for: Leveraging Physical Literacy in Schools to Enhance Meaningful Physical Activity Experiences: Mixed‐Methods Findings From an Extracurricular Intervention
Source: J Sch Health. 2026 Nov 10;96(11):e70287. doi: 10.1111/josh.70287 (PMC13750100; doi:10.1111/josh.70287)
Supplement: Supplementary file 3 [file JOSH-96-0-s003.docx]

| **Ní Chróinín et al. (2018)^[1]^, Kretchmar (2006)^[2]^, Beni et al. (2019)^[3]^** | **Ho et al. (2021)^[4]^**   - **QTPE** | **Stabick et al. (2023)^[5]^** | **Herrmann (2019)^[6]^**   - **QUALLIS** | **Guiding question** | **Category** |
| --- | --- | --- | --- | --- | --- |
| **Social interactions** (shared positive participation with others) | **Communicate ideas, feelings effectively with others** |  |  | I enjoyed moving around with the other children. | Social interactions |
| **Increased motor competence** (opportunities for learning and improved skilfulness)  **Challenge** (engagement in activities that are „just-right“) | **Learn and develop basic skills of different physical and spart activities**  **Basic motor skills** within the context of appropriate physical activities of low organization | **Challenging tasks** (focus on movement problems –experience differences in movement – learning new things) | **Physical activation**  (level – structure – feedback) | I have become better at sport. (e.g. stronger or faster) | Improvement of motor skills and abilities |
|  | **Demonstrate the basic understanding of the importance of physical activities and health.**  Demonstrate basic skills in **decision making, communication,** etc. | **Encourage reflection** (Reflection in and on movement) | **Cognitive Activation** (focussing – processing – comprehension) | I have learnt a lot about movement and my body. | Cognitive activation |
| **Delight** (sustaines pleasure or joy of significant engagement and commitment)  **Fun** (Enjoyment in the movement) |  |  | **Student orientated atmosphere**  (positive feedback, caring – support) | I looked forward to the lesson every week. | Affective approach to sport |
|  |  | **Individual support of learning** (differentiated planning of lessons – individual support) | **Lesson and classroom management**  (time usage – discipline – target clarity) | The lessons were well led. | Teaching and classroom management |
|  | At middle class level, **developing appropriate health and fitness understanding** that includes setting and achieving personal goals for healthy living |  |  |  |  |

1. Ní Chróinín, D., Fletcher, T., O’Sullivan, M. (2018). Pedagogical principles of learning to teach meaningful physical education. Physical Education and Sport Pedagogy 23 (2), S. 117-133.
2. Kretchmar, R. S. (2006). Ten More Reasons for Quality Physical Education. Journal of Physical Education, Recreation and Dance 77 (9), S. 6–9.
3. Beni, S., Fletcher, T., Ní Chróinín, D. (2019). Using features of meaningful experiences to guide primary physical education practice. European Physical Education Review 25 (3), S. 599-615.
4. Ho, W. K. Y., Ahmed, Md. D., Kukurova, K. (2021). Development and validiation of an instrument to assess quality physical education. Cogent Education 8.
5. Stabick, O., Heemsoth, T., Krieger, C., Bähr, I. (2023). Kriterien eines aktivierenden Sportunterrichts. Theoretische Verortung und empirische Fundierung aus der Sicht der Sportlehrkräfte. German Journal of Exercise and Sport Research.
6. Herrmann, C. (2019). Evaluation der Unterrichtsqualität im Sportunterricht mit dem QUALLIS-Instrument. Bewegung und Sport, 73(2), 12–17.
