# Supplementary material for: Leveraging Physical Literacy in Schools to Enhance Meaningful Physical Activity Experiences: Mixed‐Methods Findings From an Extracurricular Intervention
Source: J Sch Health. 2026 Nov 10;96(11):e70287. doi: 10.1111/josh.70287 (PMC13750100; doi:10.1111/josh.70287)
Supplement: Supplementary file 4 [file JOSH-96-0-s004.docx]

| **Item Nr.** | **Description** | **Location in the Paper  and/or Comment** |
| --- | --- | --- |
| **TITLE** | | |
| 1 | Highlight the role of PL in the title |  |
| **BACKGROUND AND DEFINITION** | | |
| 2 | Describe the relevance of PL for the target population/group/individual |  |
| 3 | Explain your conceptualization of PL and refer to a holistic definition of PL |  |
| 4 | Formulate PL-related goals/aims of your study |  |
| **ASSESSMENT** | | |
| 5a | *If quantitative:* Choose a multidimensional assessment strategy of PL and provide information about psychometric properties |  |
| 5b | *If qualitative:* Develop a qualitative method that closely aligns with PL theory and the different domains |  |
| **DESIGN AND CONTENT** | | |
| 6 | Ensure that your interventional approach is in line with PL-compatible philosophical assumptions |  |
| 7 | Mention the intervention provider(s), describe his/her/their expertise specific to PL, and any specific training given |  |
| 8 | Report in detail intervention content related to all PL domains |  |
| 9 | Explain whether and how you realized the integrative arrangement of content/techniques |  |
| 10 | Consider general guidelines for intervention reporting |  |
| **EVALUATION** | | |
| 11 | Describe how the PL intervention was accepted and/or whether it was implemented as intended (modifications, fidelity, compliance, adherence) |  |
| 12a | *If quantitative:* Report transparently how the different PL domains (and, if initially intended, other relevant outcomes such as health) were affected by the intervention |  |
| 12b | *If qualitative:* Characterize the strengths, weaknesses, and challenges of your PL intervention; the different PL domains may help you structure the analysis and results |  |
| **DISCUSSION AND CONCLUSION** | | |
| 13 | Discuss the limitations of your PL intervention, especially whether (if yes, where and why) you had to deviate meaningfully from your planned conceptualization |  |
| 14 | Break down your experiences with the PL intervention and derive sound recommendations for future studies |  |

Note: PLIRT = Physical Literacy Interventions Reporting Template; PL = Physical Literacy.

Carl, J., Barratt, J., Arbour-Nicitopoulos, K. P., Barnett, L. M., Dudley, D. A., Holler, P., Keegan, R., Kwan, M., Scurati, R., Sum, R. K.‑W., Wainwright, N., & Cairney, J. (2023). Development, explanation, and presentation of the Physical Literacy Interventions Reporting Template (PLIRT). *International Journal of Behavioral Nutrition and Physical Activity*, *20*(1), 21. https://doi.org/10.1186/s12966-023-01423-3.
